# Analytical CPG model driven by limb velocity input generates accurate temporal locomotor dynamics

**DOI:** 10.7717/peerj.5849

**Published:** 2018-10-08

**Authors:** Sergiy Yakovenko, Anton Sobinov, Valeriya Gritsenko

**Affiliations:** 1 Department of Human Performance—Exercise Physiology, School of Medicine, West Virginia University, Morgantown, WV, United States of America; 2 Department of Biomedical Engineering, Benjamin M. Statler College of Engineering and Mineral Resources, West Virginia University, Morgantown, WV, United States of America; 3 Rockefeller Neuroscience Institute, School of Medicine, West Virginia University, Morgantown, WV, United States of America; 4 Mechanical and Aerospace Engineering, Benjamin M. Statler College of Engineering and Mineral Resources, West Virginia University, Morgantown, WV, United States of America; 5 Department of Neuroscience, School of Medicine, West Virginia University, Morgantown, West Virgnia, United States of America; 6 Department of Human Performance—Physical Therapy, School of Medicine, West Virginia University, Morgantown, WV, United States of America

**Keywords:** Model, CPG, Locomotion, Steering

## Abstract

The ability of vertebrates to generate rhythm within their spinal neural networks is essential for walking, running, and other rhythmic behaviors. The central pattern generator (CPG) network responsible for these behaviors is well-characterized with experimental and theoretical studies, and it can be formulated as a nonlinear dynamical system. The underlying mechanism responsible for locomotor behavior can be expressed as the process of leaky integration with resetting states generating appropriate phases for changing body velocity. The low-dimensional input to the CPG model generates the bilateral pattern of swing and stance modulation for each limb and is consistent with the desired limb speed as the input command. To test the minimal configuration of required parameters for this model, we reduced the system of equations representing CPG for a single limb and provided the analytical solution with two complementary methods. The analytical and empirical cycle durations were similar (*R*^2^ = 0.99) for the full range of walking speeds. The structure of solution is consistent with the use of limb speed as the input domain for the CPG network. Moreover, the reciprocal interaction between two leaky integration processes representing a CPG for two limbs was sufficient to capture fundamental experimental dynamics associated with the control of heading direction. This analysis provides further support for the embedded velocity or limb speed representation within spinal neural pathways involved in rhythm generation.

## Introduction

The mechanism of spinal rhythmogenesis is an integral part of the mammalian locomotor system that fuses descending and sensory feedback signals with body dynamics (Dickinson et al., 2000). The theoretical description of this element, termed the central pattern generator (CPG), has been the focus of research with diverse aims. Previous computational studies introduced a variety of models to describe inter- and intra-limb coordination (Yakovenko et al., 2005; Schöner, Jiang & Kelso, 1990) and the rhythm generating network dynamics (Daun, Rubin & Rybak, 2009; Barnett & Cymbalyuk, 2014). Other models tested the organization of spinal interneuronal circuitry (Bashor, 1998; Rybak et al., 2006) and the dynamic interactions between the mechanical system and the CPG (Taga, Yamaguchi & Shimazu, 1991). The elusive mechanism of locomotor pattern generation remains poorly understood in the context of its regulation and integration within descending feedforward and sensory feedback pathways. One of the main obstacles is the definition of CPG’s essential function. We know that this neural element can compute control commands for the redundant musculoskeletal system (Gritsenko et al., 2016) that, in turn, shapes the activity of hierarchical neural mechanisms (Lillicrap & Scott, 2013) distributed along the neuraxis (Grillner, 1985). Moreover, the spinal motor circuits are known to accommodate rewiring in healthy operation (Vahdat et al., 2015) and injured states (Stevenson et al., 2015; Liu et al., 2017).

The computational models of CPG may help to define the role of this element within the sensorimotor hierarchy. What would be the pertinent CPG model for this task? There are multiple models, and their implementation varies in complexity mostly due to the nature of addressed problems. One of the main challenges in computational neuroscience is the choice of appropriate model complexity and the level of abstraction for the theoretical description of complex neural mechanisms. The rule of thumb for an appropriate choice of mathematical model is to match the dexterity of experimental and theoretical descriptions. For example, the experimental data representing cellular mechanisms are captured with Hodgkin–Huxley (H-H) equations that detail the observed changes in membrane properties with the nonlinear dynamics of ion channel conductances. In contrast, the network behavior is assessed most optimally with the relatively simple phenomenological rate models that approximate the details of neural spiking by their discharge rate (Sterratt et al., 2011). Recently, the CPG models with H-H formulations were applied to cross the multiscale and multilevel divide between cellular and network levels at the cost of high parametric dimensionality but describing the underlying mechanisms responsible for neural discharge (Rybak, Dougherty & Shevtsova, 2015; Danner et al., 2016).

The multiscale problem of representing input–output relationships using different physical laws and mathematical implementations to capture physical phenomena at different scales is not commonly addressed in the context of CPG models. Yet there is a long-standing history of varied techniques for simulating CPG dynamics that span physical simulations of reciprocal integrators with inhibition (Verzár, 1923), nonlinear oscillators and rate models (Patla, Calvert & Stein, 1985; Pribe, Grossberg & Cohen, 1997), and models based on spiking neurons with varied complexity of computational dynamics (Selverston et al., 2000; Rybak et al., 2006). The scope of questions addressed with these models is also surprisingly wide, e.g., quantifying the role of ionic currents shaping the bursting activity of single neurons (Kueh et al., 2016) or identifying the role of specific network elements within the CPG simulated with either the H-H models (Ausborn et al., 2017) or the rate models (Sobinov & Yakovenko, 2018).

In our previous studies using a rate CPG model, we used data-driven parameter optimization to describe locomotor phase modulation (Yakovenko et al., 2005) and then applied the inverse solutions from empirical data to identify limb speeds as the modality of computed CPG inputs (Yakovenko, 2011). Unlike in classical Marr’s top-down analysis (Marr, 1982), the CPG structure was used as a “wetware” implementation in the bottom-up analysis to identify the nature of neural computation in locomotor tasks. Similar results were also found using H-H type CPG models, i.e., the monotonic relationship between the input strength and the frequency of locomotion (Rybak et al., 2006) or limb speed (Danner et al., 2016) were identified.

Using an analytical CPG model, we have demonstrated previously that the asymmetric gait can be represented with the strengths of connections between intrinsic elements of a relatively simple bilateral CPG (Sobinov & Yakovenko, 2018). In contrast, our focus in this study was to test the prediction that the elements of a single limb CPG are sufficient for the implementation of the relationship between speed and step cycle duration. For this purpose, we derived the analytical solution for the single limb model consisting of two coupled integrators. Then, we hypothesized that the general form of the solution is consistent with the velocity command input that modulates appropriately the timing of locomotor phases. Since limb-dependent phase modulation was also implicated in the control of heading direction (Courtine, Papaxanthis & Schieppati, 2006), we used the analytical solution to demonstrate, for the first time, that single-limb velocity command signals are capable of appropriate phase modulation necessary for the control of heading direction.

## Methods

### CPG structure and function

**Figure 1:**
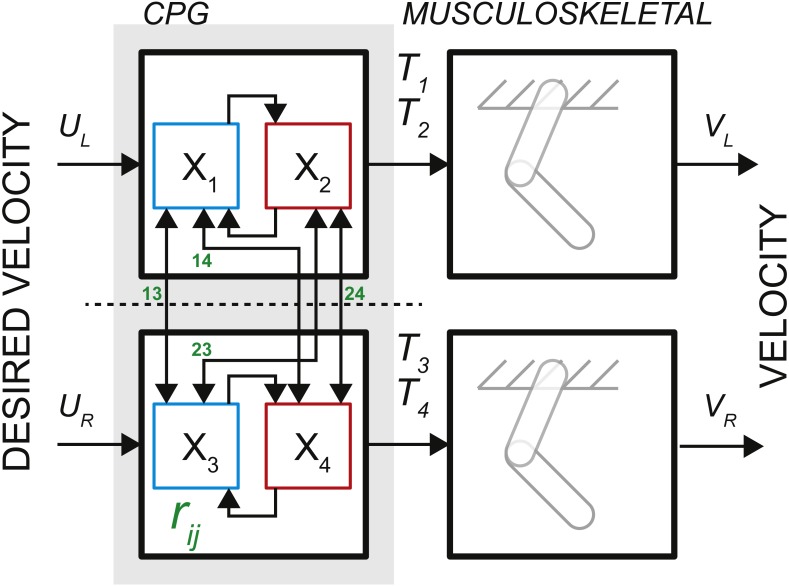
The schematic of bilateral CPG. Each locomotor phase *Ti* is generated by the transformation of low-feature inputs (desired velocity) with the intrinsic interactions between the half-centers (weights *r*_*ij*_, see Eq. (2)). The outputs in the form of phase durations define the pattern of flexor and extensor motoneurons responsible for the activity of muscles during swing and stance for each limb.

The observations of neural activity in the absence of descending signals or sensory feedback led T.G. Brown to formulate the principle of intrinsic rhythmogenesis of spinal networks, the half-center oscillator hypothesis (Brown, 1911). Brown posited that “…*the centres are paired, and that each pair consists of antagonistic opposites*.” The intrinsic rhythmogenesis opposed the established view that the locomotor pattern is generated and shaped only by supraspinal and sensory feedback pathways. The bilateral CPG model in Fig. 1 was developed from a numerical model of a single-limb oscillator to describe phase dominance in fictive cat locomotion, which is a type of experimental behavior with diminished sensory contribution (Yakovenko et al., 2005). This model controlling two limbs consisted of two dedicated oscillators made of two reciprocally coupled half-center elements (gray area in Fig. 1). It can generate bilateral rhythm using the interactions within and between the half-center elements. Only the rhythm generating mechanism is captured by this feedforward rate model with time-varying inputs. The pattern formation mechanism responsible for the generation of motoneuronal input signals can be computationally decoupled from the temporal dynamics of rhythm generation (McCrea & Rybak, 2008).

**Figure 2:**
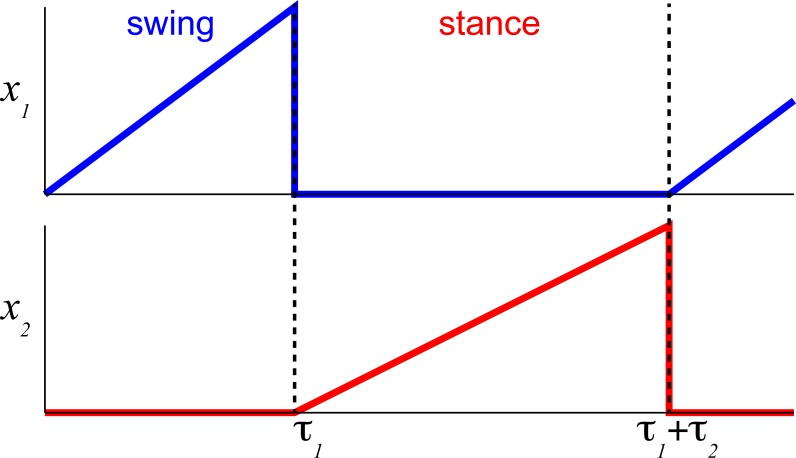
The temporal schematic of two reciprocal states with integration and resetting. The integration process in flexor half-center (blue) described by Eqs. (3) and (7) is reset to 0 (minimal value) after reaching 1 (maximal value) and the reciprocal extensor state (red) is initiated with the same state-switching constraint.

The process of controlling locomotor phase durations is based on the ability of the network to integrate inputs until reaching a critical threshold causing a phase resetting within the CPG network, Fig. 2. We have previously developed the bilateral model (Yakovenko, 2011; Sobinov & Yakovenko, 2018) and describe it in brief here. The model was expressed as the system of differential equations consisting of two parts in Eq. (1): (i) the largely extrinsic signals (right side) and (ii) the intrinsic interactions (left side). The offset term (*x*_0_) could combine both intrinsic and extrinsic influences on the background excitability of spinal cord. The bilateral CPG model consists of a system of differential equations for four intrinsic states ($x={ \left( {x}_{1},{x}_{2},{x}_{3},{x}_{4} \right) }^{T}$) that represent flexor and extensor locomotor phases for each limb:

(1) \begin{eqnarray*}\dot {x}-{G}_{x}^{UL}x={x}_{0}+{G}_{u}u\end{eqnarray*}

where *Gu* matrix represents gains of input signals *u*, *x*_0_ are constant offset values, ${G}_{x}^{UL}$ matrix represents the strength of unilateral connections between the CPG half-centers (shown as arrows with weights *r*_*ij*_ in Fig. 1, the connections across the midline were removed). ${G}_{x}^{UL}$ matrix has the following form:

(2) \begin{eqnarray*}{G}_{x}^{UL}=I\ast {r}_{\mathrm{ leak}}\end{eqnarray*}

where *I* is the identity matrix, *r*_leak_ is the constant that determines intrinsic state-dependent feedback. This parametric transection effectively decouples the control of left and right limb. However, the bilateral coupling in this model could still be achieved through the common descending drive (as demonstrated for the heading direction control, below).

The internal states are limited to positive values with the switching threshold set to 1. Only one state from a pair, 1–2 (Fig. 2), is set to be active *x* ∈ (0, 1] to impose the reciprocal relationship between half-centers. This implementation assumes robust reciprocity between antagonistic states and enforces zero overlap between them. The single limb CPG would consist only of two reciprocal states ($x={ \left( {x}_{1},{x}_{2} \right) }^{T}$).

Even this simple model had many parameters that were largely undefined. Using an error-driven search algorithm in our previous study (Yakovenko, 2011) we found a set of optimal parameters (Table A1). These parameters were resolved by the minimization of the objective function with terms related to the errors in simulating swing and stance phases and the rate of their modulation for different overground speeds (Goslow, Reinking & Stuart, 1973; Halbertsma, 1983).

## Results

The relationship between step cycle duration and the input “drive” to the analytical model was investigated in two complimentary solutions that rely on different assumptions: (i) the assumption of constant integration rate in a single limb model of CPG, and (ii) the expansion of function with the common Taylor series method.

### Solution using constant rate assumption

First, let us express explicitly all the term in Eq. (1) to describe only flexor and extensor states controlling a single limb. Here, *x*_1_ and *x*_2_ are the reciprocal state variables as shown in Fig.  1. The system of equations can then be stated as:

(3) \begin{eqnarray*} \left\{ \begin{array}{@{}l@{}} \displaystyle {\dot {x}}_{1}={x}_{01}+{g}_{u1}u+{r}_{\mathrm{leak}}{x}_{1} \\ \displaystyle {\dot {x}}_{2}={x}_{02}+{g}_{u2}u+{r}_{\mathrm{leak}}{x}_{2}. \end{array} \right. \end{eqnarray*}

Since *r*_leak_ is a small negative number (Table A1) the rate of state ($\dot {x}$) can be further approximated without this term using phase duration quantities as the difference of states for a given phase duration, i.e., the inverse of phase duration. Even for the time-variable input (*u*), the rate of state for a full phase duration can be simplified as:

(4) \begin{eqnarray*}\dot {x}= \frac{max-min}{\tau } = \frac{1}{\tau } .\end{eqnarray*}

Figure 2 shows an example for this formulation based on Eq. (1) for a single limb with the assumption of the constant rate of integration (*g*_*u*1_ and *g*_*u*2_ are scalars, as in our previous studies). Each state (*x*_1_ and *x*_2_) integrates an input (*u*) only when active. The integration rate per step cycle can then be stated as in Eq. (4). This formulation is possible due to the removal of midline crossing connections (green in Fig. 1) between CPG states that complicate the relationship. Then the expression for cycle duration can be described as a sum of the antagonistic phases in the simplified system, Eq. (5):

(5) \begin{eqnarray*}{T}_{c}={\tau }_{1}+{\tau }_{2}= \frac{1}{{\dot {x}}_{1}} + \frac{1}{{\dot {x}}_{2}} = \frac{{\dot {x}}_{1}+{\dot {x}}_{2}}{{\dot {x}}_{1}{\dot {x}}_{2}} .\end{eqnarray*}

Since the cycle duration, *Tc*, is a constant for a given constant input (*u*), the only time-varying variables are the states of the system, *x*_1_ and *x*_2_. In phase transition points, at *t* = *τ*_1_ or *t* = *τ*_1_ + *τ*_2_, *x*_1_ and *x*_2_ are zero or a small value close to zero. We can further expand this equation with Eq. (3) and simplify it to all the known terms:

(6) \begin{eqnarray*}{T}_{c}= \frac{{x}_{01}+{x}_{02}+ \left( {g}_{u1}+{g}_{u2} \right) u}{({x}_{01}+{g}_{u1}u)({x}_{02}+{g}_{u2}u)} = \frac{a+bu}{\tilde {a}+\tilde {b}u+\tilde {c}{u}^{2}} \end{eqnarray*}

where the step cycle duration is expressed as a function of input (*u*) and all parameters *a*, *b*, $\tilde {a}$, $\tilde {b}$, $\tilde {c}$ are constants determined by the coefficients in the system of equations Eq. (3).

### Solution using Taylor series

The same solution Eq. (6) was found by integrating the differential equations (3) between 0 and *t*. For this, Eq. (3) can be rewritten with the assumption of independent limb control:

(7) \begin{eqnarray*}\dot {x}-rx={x}_{0}+{G}_{u}u\end{eqnarray*}

where variables are as defined for Eq. (1), and *r* = *r*_leak_. Note that the right-hand side can be assumed to be time-independent for constant input (*u*) and this type of equations has a general solution of the form *e*^*kx*^. The left side of the above equation can be expressed as

(8) \begin{eqnarray*}{ \left( x{e}^{-rt} \right) }^{{^{\prime}}}=\dot {x}{e}^{-rt}-rx{e}^{-rt}=(\dot {x}-rx){e}^{-rt}.\end{eqnarray*}

Hence, Eq. (7) can be integrated and evaluated between 0 and *t* using

(9) \begin{eqnarray*}& & { \left. \left( x{e}^{-rt} \right) \right\vert }_{0}^{t}=\int \nolimits \nolimits _{0}^{t}({x}_{0}+{G}_{u}u){e}^{-rt}dt\end{eqnarray*}

(10) \begin{eqnarray*}& & x \left( t \right) {e}^{-rt}-0= \frac{{x}_{0}+{G}_{u}u}{-r} ({e}^{-rt}-1)\end{eqnarray*}

(11) \begin{eqnarray*}& & x \left( t \right) = \frac{{x}_{0}+{G}_{u}u}{r} ({e}^{rt}-1).\end{eqnarray*}

The exponential function can be further expanded with Taylor series and some components can be dropped since *r* is a number close to zero, so that *rt* ≈ 0 in the expansion:

(12) \begin{eqnarray*}x \left( t \right) \approx \frac{{x}_{0}+{G}_{u}u}{r} \left( 1+rt+\ldots -1 \right) \approx ({x}_{0}+{G}_{u}u)t.\end{eqnarray*}

Then, the full phase of each integrated state is

(13) \begin{eqnarray*}t= \frac{1}{{x}_{0}+{G}_{u}u} .\end{eqnarray*}

Finally, the full cycle duration consisting of two reciprocal phases (*t*_1_ + *t*_2_) has the same form as Eq. (6)

(14) \begin{eqnarray*}{T}_{c}={t}_{1}+{t}_{2}= \frac{a+bu}{\tilde {a}+\tilde {b}u+\tilde {c}{u}^{2}} \end{eqnarray*}

where *a*, *b*, $\tilde {a}$, $\tilde {b}$, $\tilde {c}$ are constants defined by the examination of algebraic terms from Eq. (13).

### Validation

**Figure 3:**
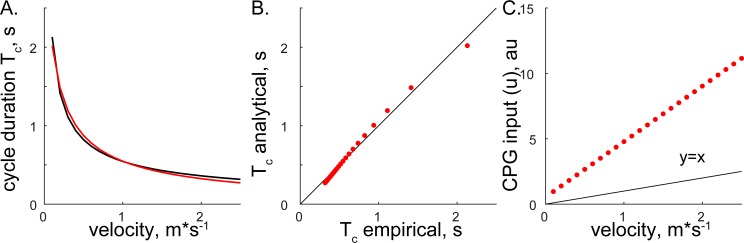
The comparison of analytical and empirical values. (A) The solution of cycle durations is shown for both the analytical (red) and empirical (black) values. (B) The analytical cycle durations (*Tc*) are plotted as a function of empirical *Tc* (*R*^2^ = 0.9946, *p* < 0.001). (C) The relationship between input signals and empirical forward velocity.

**Figure 4:**
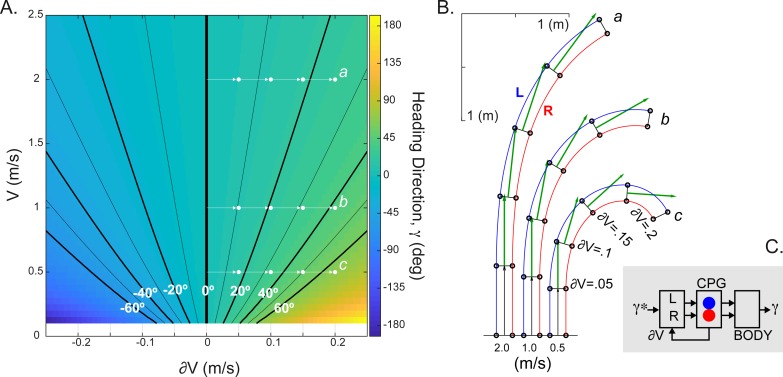
The simulated relationship between CPG inputs (limb speeds) and the heading direction. (A) The change in the heading direction is shown as a function of two parameters—mean speed and limb speed differential. (B) Examples of asymmetrical walking trajectories simulated for the ranges marked (*a-c*) in (A). The heading direction (green) was scaled with the mean stride length in five simulated steps. (C) Schematic summarizing the heading direction control based on the velocity command hypothesis. The desired heading direction (*γ*∗) can automatically generate the CPG speed commands appropriate for steering body (*γ*).

Both methods converged on the same form, Eqs. (6) and (14), supporting the consistency of solutions with different assumptions. The relationship between step cycle duration and CPG input (*Tc* and *u*) is of the form *T*_*c*_ = *au*^−*b*^. This simple analytical solution has a similar form to the phenomenological relationship between cycle duration and the velocity of overground forward progression *T*_*c*_*=0.5445V*^−0.5925^ (Goslow, Reinking & Stuart, 1973). Figure 3 shows the comparison of solutions with our analytical and the previous phenomenological model for the step cycle duration and velocity values. The simulated *T*_*c*_ data values were calculated with Eq. (7) using optimal parameters and *u* values selected with the regression equation *u* = (*V* + 0.1272)∕0.2357 (from Fig. 4 in our previous work (Yakovenko, 2011)) and plotted in Fig. 3C. The analytical solution (red) for leg speed was closely related to the empirical curve (*black*) calculated with the phenomenological functions that were calculated as the best-fit expressions for the experimental measurements (Goslow, Reinking & Stuart, 1973; Halbertsma, 1983) (Fig. 3A). Since the swing duration remains nearly constant as a function of either step cycle duration or the velocity of forward progression in cats (Halbertsma, 1983; Frigon et al., 2014), it can be approximated as a constant (≈0.25 s). Then, the stance duration is the same as in Eqs. (6) and (14) with the negative constant. This relationship may not be preserved for gaits with the large differences in limb speeds as those used in split-belt experiments (D’Angelo et al., 2014) and may require the consideration of bilateral inputs as in our previous study (Sobinov & Yakovenko, 2018). The analytical and empirical step cycle durations were highly correlated (Fig. 3B) for the linear relationship between CPG inputs (*u*) representing scaled forward velocity values (Fig. 3C).

The implementation of CPG with the limb speed inputs is expected to generate spatiotemporal step modulation appropriate for the locomotion on a curved path. We used the following equation to compute the relationship between the heading direction (*γ*) and limb speeds (*V*_*R*_, *V*_*L*_) : *γ* = *Tc*(*V*_*R*_−*V*_*L*_)∕*W*, where *W* is the interlimb step width (≈0.15 m in cat) and Tc is the step duration from Eq. (6). This equation was previously derived for the experimental and theoretical kinematics of locomotion along a curved path (Courtine, Papaxanthis & Schieppati, 2006; Sobinov & Yakovenko, 2018). Figure 4A shows the monotonic relationship with extremes occurring for large interlimb speed difference (∂V) at slow speeds. This range may be consistent with the “spin turning” when body spins around a supporting limb (Hase & Stein, 1999). Examples of simulated kinematics for three speeds (a, b, c = 0.5, 1, 2 m/s) with increasing interlimb speed differences in each consecutive step are shown in Fig. 4B. The green vector indicating the heading direction demonstrates the dependency not only on the interlimb speed difference (∂V), but also the overall magnitude of body’s velocity (*V*). The parsimonious analytical CPG model that includes computations for two limbs can generate steering.

## Discussion

Here, we have investigated an extreme example of the structural feedforward rate model with time-varying inputs and its ability to capture general CPG function. We have developed an analytical solution for a reduced CPG model to test if the basic structure of reciprocal interactions between integrating and leaky network elements can generate appropriate input–output relationship between limb speed and locomotor cycle duration. The analytical solution of the reduced CPG model recreated the empirical data very closely, despite model simplicity and assumptions in deriving the solution. This was not clear *a priori*.

The minimalistic implementation of CPG required significant assumptions about morphology and function in the model. Both, flexor and extensor half-centers were assumed to be capable of generating rhythm based on the reciprocity of two integrating circuits. The ability for rhythmogenesis of each half-center is the current consensus among multiple groups (see reviewed in Frigon, 2017), but it has been under some scrutiny, see discussion of “swing-phase” CPG below. In the model, the switching to the antagonistic phase is triggered by the state signal crossing the threshold (*x*_*i*_ = 1). The process responsible for maintaining activity in one phase is similar to the dynamics arising from the slowly inactivating persistent sodium current in CPG models using H-H dynamics.

The dynamical rate models describing the single-limb CPG are sufficient for the description of the relationship between the desired speed and the locomotor phases. The main advantage of simple models is that their parameters can be accurately scaled using empirical data on the timing of locomotor phase transitions. While we have used data from observations of cat locomotion, the general functional homology of the CPG mechanism has been demonstrated in other mammals, including humans (Lam & Yang, 2000; Musselman & Yang, 2007; Dominici et al., 2011). As further anatomical studies detailing the organization and wiring of neurons become available for mammalian CPG (Kiehn, 2016), the inclusion of these details in models is generally left to the intuition. H-H spike-generating models of CPG require multiple estimated parameter values that are often difficult to validate in numerical simulations. These models provide insight into the realistic control challenges and reveal tentative explanations of experimental discrepancies. For example, the discrepancy between the observation of both extensor and flexor phase dominance in locomotor patterns generated by adaptable flexor- and extensor- driven CPG as opposed to only the flexor-driven CPG (see review Duysens, De Groote & Jonkers, 2013) can be reconciled with the consideration of available functionality within underlying single-cell and network dynamic elements (Ausborn et al., 2017). A subset of plausible mechanisms selected from the plethora of unexplored parametric relationships can explain multiple observed states, and other alternative mechanisms generating similar outcomes may exist within the same models.

The evidence of underfitting of experimental data by simple models should be the main motivation for the inclusion of additional terms within theoretical representations. As we have observed in a relatively complex dynamical rate model simulating asymmetric bilateral locomotion (Sobinov & Yakovenko, 2018), the same low-dimensional output can be produced by several alternative parameter configurations. What region of the parameter space, which is nine-dimensional for a bilateral rate model, is physiological remains to be established. The potential of dynamical rate models to simulate brain functions also remains an open question. Their utility was demonstrated in a series of studies of motor cortical processing spanning reaching movements and motor learning (Churchland et al., 2012; Gilja et al., 2012; Kao et al., 2015; Sussillo et al., 2015). Our finding suggests that dynamical rate models solve the problem of transforming high-level commands to CPG by capturing empirical observations of temporal phase relationships.

All parameters in the parsimonious single limb CPG model can be robustly constrained by the corresponding empirical observations. In this model, the inputs are isolated and identified as velocity-dependent based on the observed outputs. For example, it is sufficient to measure the phase and cycle relationship to identify the scalar gain and the offset for each half-center integrator. The excluded connectivity within the CPG model representing propriospinal commissural pathways in the lumbosacral enlargement removes the network rhythmogenic flexibility that may represent different behavioral states intrinsically (Ausborn et al., 2017; Sobinov & Yakovenko, 2018). For example, a simulation using a high-dimensional parameter space model developed with H-H formalism has demonstrated that the interneurons crossing the midline may provide left–right limb coordination (Shevtsova et al., 2015). The reduction of the high-dimensional parametric space reduces inevitably the behavioral repertoire but increases the model robustness and gains the simple expression of the underlying system characteristics. While the utility of simple models can be challenged for problems that require the examination of intricate structural or functional details, the use of simple models conforms to George Box’s truism that “all models are wrong but some are useful” (Box, 1979). The dimensionality of both inputs and outputs in this model’s dynamical transformation is equivalent, and all parameters are readily definable by the statistics of observations. Could these model parameters be related to physiological variables? We have previously speculated (Yakovenko et al., 2005) that the “offset” parameter (x0) corresponds to the overall excitation of cells involved in rhythm generation. For example, MLR stimulation or sensory feedback could modify this excitability level. The “gain” parameter (g) may correspond to the required range of variation in the simple oscillator. The dynamic rate oscillator models can then disentangle relative contributions of multiple converging pathways for the regulation of excitability or the dynamic range of recruitment within the timing-generating populations of neurons.

The presented solution is based on the analysis of a single limb controller. How does this apply to the behaviors with the interlimb contributions? In a quadruped, the CPG is a network of all four limb controllers that generate patterns with the inputs of all its elements. The analyses of locomotor patterns in split-belt locomotion, when fore- and hind- limbs or left and right limbs were decoupled and allowed to move at different speeds, support the idea that forelimb and hindlimb CPGs are similarly organized without midline asymmetries (D’Angelo et al., 2014). The upper and lower limb CPG networks have been proposed to monitor and to integrate sensory inputs with the ongoing rhythmic activity both in cats and also in humans (Duysens & Van de Crommert, 1998). For example, the cutaneous inputs are similarly modulated in lower limbs during locomotion and in upper limbs during rhythmic, cyclical arm tasks (Zehr & Kido, 2001). The similarity in the structure of the upper and lower limb controllers and their symmetricity across the midline corroborates the idea that the understanding of single limb CPG dynamics is central to the description of inter limb coordination and sensorimotor integration. Thus, this model may be adapted in the future studies to capture, at least partially, upper-limb dynamics in rhythmic movements.

Our results have shown that a simple CPG model driven by limb velocities captures the behavior of steering during gait. In studies where subjects were asked to walk on curvilinear paths (Hase & Stein, 1999; Courtine, Papaxanthis & Schieppati, 2006), both amplitude and timing in leg and trunk muscles were modulated. This supports the idea that the descending command interacts directly with the CPG circuitry to change the heading direction and to allow the locomotion along the curvilinear path. The control of turning during locomotion has been described in the context of controlling subject’s center of mass (Patla, Adkin & Ballard, 1999). The dynamics of this problem is typically defined by the model of inverted pendulum (Hof, 2008), which is traditionally used as the basis of neural transformation responsible for the locomotor rhythmogenesis and mechanical stability (Full & Koditschek, 1999; Full et al., 2002). The neural circuitry of CPG mechanism coupled to the mechanical dynamics of limbs is thought to anticipate mechanical requirements, a phenomenon termed neuromechanical tuning (Taga, Yamaguchi & Shimazu, 1991; Prochazka & Yakovenko, 2007). Thus, it is logical to hypothesize that the anticipated heading direction signal is processed by the CPG network. However, the operation of this pathway may be limited to the “step turning”, which has no abrupt trunk rotation, as opposed to “spin turning”, which may require stopping the axial leg on the inside of a turn (Hase & Stein, 1999). The step turning is generally stable with wider step width and does not disrupt the gait rhythm. Thus, the limb speed driven CPG may mediate turning through a step turning strategy (Fig. 4). In the task where a subject walks on the same curved path with different limb speeds, the simulations predict an increase in interlimb speed with the increasing velocity—moving along the same heading direction line in Fig. 4. Similar increase in the limb stride length asymmetry can be seen in the kinematics of human curved locomotion (see Fig. 4 in Orendurff et al., 2006).

The desired heading direction may be expressed within limb speed commands that descend to the CPG. Similar to our previous study (Yakovenko, 2011), the simple analytical formulation of the relationship between the heading direction and the CPG inputs can be analyzed bottom-up, where a system producing body reorientation during locomotion is also driven by the desired heading direction originating from the higher levels of the visuomotor pathway. The support for the expression of desired heading direction comes from the observations of anticipatory head orientation in humans walking on curved paths and the existence of dedicated visuomotor cells tuned to the head orientation. The orientation of head relative to the desired locomotor direction (similar to γ* term in Fig. 4C) may enable the repositioning of body relative to its frame of reference associated with the ongoing forward progression (Hollands, Sorensen & Patla, 2001). Neurons encoding selectively the head orientation, termed “head direction cells”, have been found in the visuomotor and navigation-related pathways of several mammals (Taube, Muller & Ranck, 1990a; Taube, Muller & Ranck, 1990b; Knierim, Kudrimoti & McNaughton, 1995; Robertson et al., 1999; Sargolini et al., 2006) and simulated in models (Zhang, 1996; McNaughton et al., 2006). The head direction has been shown to influence selection of limb movements (Dancause & Schieber, 2010) and to anticipate the turning in walking on straight and curved paths (Hicheur, Vieilledent & Berthoz, 2005). In the system with the desired heading direction control, the left–right limb coordination would be automatically generated within the pathways converging on the CPG network.

The current model implementation has both temporal and spatial limitations. It has been validated for the temporal modulation of step cycle duration within the range of walking speeds. However, it may not extend to other locomotion types where stance is shorter than swing, i.e., running. In addition, since the dynamics of single limb CPG model is largely dominated by the modulation of stance phase, the phase modulation has been simplified to the examination of only step cycle duration where *Tcycle* = *Tstance + const*. This is supported by the observations that swing phase remains nearly constant over a wide range of walking speeds (Halbertsma, 1983; Frigon, Thibaudier & Hurteau, 2015). However, the phase duration curve of flexor phase is not constant in other types of behaviors, e.g., in the “flexor dominant” type of fictive locomotion (Yakovenko et al., 2005). The speed-related increase in the exerted muscle force is expected to be matched with the increase in the order and size of recruited muscle motor units in accordance with Hennemann’s size principle (reviewed in Taylor, 1978). This quadratically increasing signal has not been represented in the current model because this implementation captures whole limb behavior and not the patterning of individual muscles, which can be achieved with the method of Patla, Calvert & Stein (1985). In neuromechanical simulations using the single-limb analytical implementation, the velocity-dependent recruitment can be added to the CPG model as the direct command from the descending pathways (velocity signal) to muscle activation (Prochazka & Ellaway, 2012) or as a transformation from the inverse of the corresponding speed-dependent phase duration. In models without this transformation, the speed-dependent increase in the recruitment of muscles can also be compensated by muscle properties and proprioceptive feedback dependent on muscle dynamics. Using a neuromechanical simulation of cat locomotion in our previous study (Yakovenko, Gritsenko & Prochazka, 2004), we showed that the low ankle extensor forces at high locomotor speeds alter gait kinematics forcing ankle extensors to operate at longer lengths, which, in turn, increases both force generation and increases the stretch reflex contribution from Ia and Ib primary afferent pathways.

The description of mechanisms responsible for the coordination of phasic activity during locomotion may be necessary for the development of stroke and spinal cord injury repair and rehabilitation strategies (Thompson, 2012). The basic mechanistic description of CPG is critical for the development of robotic and clinical applications that take advantage of this element, and it is essential for the functional understanding of hierarchical descending and sensory feedback pathways projecting to it. The fundamental dynamical form of CPG mechanism and its validation in locomotion with different velocities opens a robust alternative to computationally intensive models.

## Conclusion

The analytical solution demonstrates that the linear relationship between forward velocity or limb speed and the CPG model input is an intrinsic property of reciprocal organization between two half-center oscillators. Moreover, there is a good correspondence between the form of analytical solution and the previous empirical description of this relationship. The existence of rhythmogenic neural networks with the reciprocal inhibition makes it possible to use gross signals, i.e., limb velocity, to specify the nonlinear regulation of locomotor phases. In addition, this model can describe steering control as the CPG-mediated transformation from the internal representation of desired heading direction in terms of limb speeds to the executed change in the step cycle of each limb. Further theoretical description of CPG may provide tools for intelligent prosthetics and the quantitative metrics of locomotor disabilities.

**Table A1:** Optimal CPG parameters from Yakovenko (2011).

Parameter	*x*_01_	*x*_02_	*g*_1_	*g*_2_	*r*_leak_
Value	−0.0007	2.4256	0.6203	0.4882	−0.0094

##  Supplemental Information

10.7717/peerj.5849/supp-1 Supplemental Information 1 Matlab code to generate Fig. 3

## Additional Information and Declarations

### Data Availability

The following information was supplied regarding data availability: Yakovenko, Sergiy (2018): PeerJ2018. figshare. Code. https://doi.org/10.6084/m9.figshare.6955277.v1.
